# Clinical and Therapeutic Implications of BCAAs Metabolism during Chronic Liver Disease in Humans: Crosstalk between Skeletal Muscle and Liver

**DOI:** 10.3390/muscles3010008

**Published:** 2024-03-04

**Authors:** Maria Camila Trillos-Almanza, Magnolia Martinez-Aguilar, Johanna C. Arroyave-Ospina, Frederike van Vilsteren, Hans Blokzijl, Han Moshage

**Affiliations:** 1 Department of Gastroenterology and Hepatology, University Medical Center Groningen, University of Groningen, P.O. Box 30.001, 9700RB Groningen, The Netherlands; m.c.trillos.almanza@umcg.nl (M.C.T.-A.); l.m.martinez.aguilar@umcg.nl (M.M.-A.); h.blokzijl@umcg.nl (H.B.); 2 Gastroenterology and Hepatology Research Group, Faculty of Medicine, University of Antioquia, Street 70 No. 52-21, Medellin 0500120, Colombia; johanna.arroyave@udea.edu.co

**Keywords:** amino acids, branched-chain, chronic liver disease, muscle–liver crosstalk, BCAA supplementation

## Abstract

This comprehensive review focuses on the dynamics of branched-chain amino acids (BCAAs) metabolism and its clinical implications in chronic liver disease, with emphasis on the emerging concept of muscle–liver crosstalk. BCAAs, indispensable for protein synthesis and metabolic pathways, undergo unique tissue-specific processing in skeletal muscle and liver. The liver, responsible for amino acid metabolism, plays a distinctive role in sensing BCAAs catabolism, influencing glucose regulation and contributing to the systemic metabolism of BCAAs. Within the context of chronic liver disease, compromised liver metabolism becomes evident through amino acid abnormalities, particularly in the decrease of the Fischer ratio (BCAAs/aromatic amino acids concentrations in plasma). This reduction becomes important in assessing the severity of liver dysfunction due to its associations with adverse outcomes, including increased mortality and complications related to the liver disease. BCAAs supplementation, as explored in this review, emerges as a promising avenue, displaying positive effects on skeletal muscle mass, strength, and overall nutritional status in cirrhosis management. Understanding this interplay offers insights into therapeutic strategies for chronic liver diseases, exploring the way for precision interventions in clinical practice.

## 1. Introduction

The branched-chain amino acids (BCAAs) Isoleucine (Ile), Leucine (Leu) and Valine (Val), represent around 35% of the total twelve essential amino acids in most mammals with a relative abundance of approximately 1.6:2.2:1.0 (Val:Leu:Ile), with Leu being the most abundant and Ile the least abundant [1]. BCAAs are branched, small and hydrophobic molecules, that are used for protein synthesis [2] and derived from nutrition, since they cannot by synthesized by the body itself. In general, BCAAs share metabolic routes and they are usually present in the same dietary sources and therefore metabolized together, which is also the reason why they are usually studied in combination [3]. However, it is important to highlight their diverse biological effects, a subject that will be explored in this review.

BCAAs are not only essential substrates for protein synthesis but also regulate energy metabolism, for example, by contributing to gluconeogenesis and lipid metabolism [4]. For instance, in muscle, BCAAs provide non-specific carbons as a source for oxidation during energy production, yielding energy more efficiently than glucose [5]. In addition, BCAAs stimulate protein synthesis by enhancing mRNA translation and by favoring protein turnover [6].

The liver is responsible for BCAAs oxidation using metabolic BCAAs coming from skeletal muscle (and other tissues), to maintain protein turnover, amino acid levels and energy homeostasis (e.g., gluconeogenesis), among others [7]. Whereas most amino acids are metabolized in the liver, skeletal muscle plays a pivotal role in BCAAs metabolism, actively participating in their utilization and significantly impacting diverse physiological processes. Skeletal muscle has a high activity of the enzyme branched-chain aminotransferase 2 (BCAT2), the first enzyme involved in BCAAs catabolism which is barely expressed in the liver [8].

Skeletal muscle protein synthesis and glucose uptake are stimulated by BCAAs through the promotion of the translocation of glucose transporters to the plasma membrane [9], while proteolysis is suppressed by BCAAs [10]. Additionally, BCAAs activate metabolic signaling pathways like the mTOR and PI3K-Akt pathways, ultimately leading to insulin resistance [4,11]. Recent studies suggest that BCAAs have a role in glucose homeostasis and it has been reported that BCAAs plasma levels correlate with obesity, glucose intolerance, insulin resistance and increased risk of developing type 2 diabetes [12]. In addition, it has been reported that the liver, as a regulator of BCAAs catabolism, plays an important role in the interplay between BCAAs metabolism and energy homeostasis [13]. Moreover, defective BCAAs catabolism results in impaired glucose metabolism [14].

It has also been suggested that BCAAs might be involved in the pathophysiology of chronic liver diseases (CLD). Impairment of BCAAs catabolism has been associated with liver fibrosis [15,16]. A negative correlation between fibrosis stage and plasma concentrations of BCAAs has been described, primarily attributed to the decrease in serum BCAAs levels to compensate for the impaired hepatic urea cycle [15]. BCAAs catabolism alterations might be mainly influenced by the suppression of the enzymatic activity of the first two enzymes in their catabolic pathway, BCAAs aminotransferase (BCAT) and branched-chain α-keto acid dehydrogenase (BCKD). Therefore, these enzymes may be suitable therapeutic targets for the prevention of CLD [17,18]. The molar ratio of BCAAs residues (Leu, Ile and Val) to aromatic amino acid (AAA) residues (Tyr, Trp and Phe) is known as the Fischer ratio. A low Fischer ratio, indicative of disrupted amino acid metabolism, is associated with the development of hepatic encephalopathy (HE), sarcopenia and hepatocarcinogenesis [18] and increased mortality in advanced CLD [19]. The Fischer ratio serves as a valuable predictor of adverse clinical events, emphasizing its importance in assessing the severity of liver dysfunction and guiding therapeutic strategies. Furthermore, BCAAs supplementation is also effective against fibrosis by downregulating hepatic stellate cell activation and TGF-B signaling as a consequence of mTORC1 activation [15].

The emerging concept of skeletal muscle–liver crosstalk holds significant importance in understanding the clinical implications of BCAAs metabolism. This concept highlights the intricate communication between skeletal muscle and the liver in regulating BCAAs metabolism and overall metabolic homeostasis. By recognizing the dynamic relationship between these two organs, clinicians can better understand how alterations in BCAAs metabolism may impact various physiological processes and contribute to the pathogenesis of metabolic disorders, including CLD. In this review, we will discuss the relation between muscle and liver in BCAAs metabolism, with special emphasis on adults with CLD. 

## 2. Metabolism of BCAAs: Interplay between Skeletal Muscle and Liver

### 2.1. BCAAs Transamination

**Figure 1:**
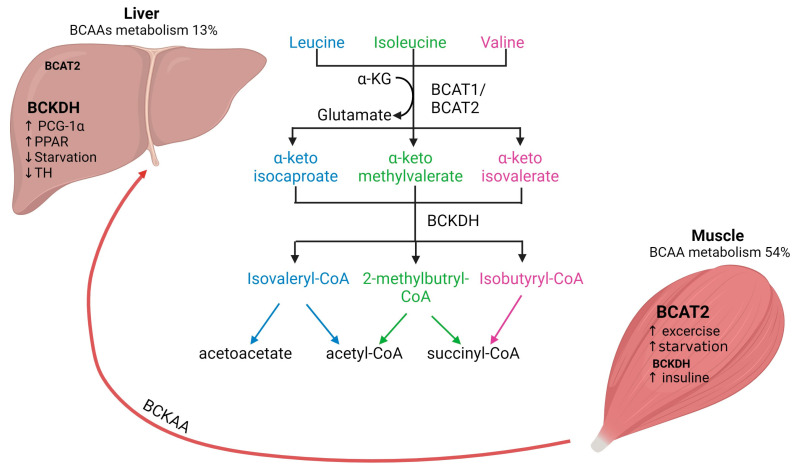
Interplay of BCAAs metabolism between human skeletal muscle and liver. Arrows represent the metabolic steps involved in the conversion and utilization of BCAAs. BCAAs, branched-chain amino acids; BCAT, branched-chain aminotransferase; BCKDH, branched-chain alpha-keto acid dehydrogenase. Figure created with Biorender.com (accessed on 13 January 2024).

Mammals cannot synthesize BCAAs, but bacteria, fungi and plants can and use threonine (for Ile) and pyruvate (for Val and Leu) as precursors [1]. In general, BCAAs account for approximately 15% of the total amount of amino acids in human muscle protein and they are the main nitrogen source for glutamine and alanine synthesis in skeletal muscle tissue [2]. The first step in BCAAs catabolism, which occurs in all organisms, is the (reversible) transamination to branched-chain α-ketoacids (BCKAs) by mitochondrial BCATs (Figure 1) [17].

There are two isoforms of BCAT: the cytosolic isoform BCAT1, with expression restricted to the brain, ovary and kidney, and the mitochondrial isoform BCAT2, found in several tissues, including skeletal muscle, kidney, brain cortex, heart, subcutaneous adipose tissue, stomach, colon, ileum, and liver [17,20]. Skeletal muscle has the highest activity of BCAT2, contributing to around 65% of BCAAs transamination compared to the liver, which accounts for only 3.8% of BCAAs transamination in human tissues (Figure 1) [2]. The critical role of BCAT2 in muscle protein synthesis and regulation of muscle protein degradation was demonstrated using BCAT2 knockout mice. These mice are leaner and smaller in size and demonstrate increased muscle protein degradation compared to wildtype mice [21]. Furthermore, it has been shown that BCAT2 expression is regulated by exercise: BCAT2 protein expression is increased by endurance training compared to resistance training, but levels are normalized again after 3 days of repetitive exercise training [22].

### 2.2. BCAAs Oxidation

The second step of BCAAs catabolism is oxidative decarboxylation (irreversible) catalyzed by the BCKDH to produce branched-chain acyl-CoA esters (Figure 1) [17]. The activity of the BCKDH enzyme is high in the liver, brain, and kidney but low in skeletal muscle [7]. Oxidative decarboxylation by BCKDH is performed mainly in the liver using BCKAs produced in the skeletal muscle, which are imported into the liver [23], implying an interplay between skeletal muscle and liver in BCAAs metabolism. However, overall skeletal muscle accounts for 54% of total BCAAs catabolism compared to only 13% in the liver [2]. The final step involves the catabolism of the branched-chain acyl-CoA esters, which differs for each BCAAs (Figure 1). Leu is converted into acetyl-CoA and acetoacetate production (ketogenic), Val into succinyl-CoA (glucogenic) and Ile into acetyl-CoA and succinyl-CoA. All these metabolites feed into the citric acid cycle (TCA) contributing to energy generation [1]. Furthermore, BCAAs conversion to BCKAs in skeletal muscle produces glutamate, which is combined with pyruvate for the production of alanine and released for its transport to the liver. Alanine is further used as a substrate for glucose generation through gluconeogenesis and, finally, this glucose is taken up by the skeletal muscle for energetic metabolism [24,25]. The interaction between skeletal muscle and liver by BCAAs trafficking is necessary for energy homeostasis.

The regulation of BCAAs is not completely understood yet. BCAT activity is physiologically upregulated with exercise and food intake but downregulated by starvation. In skeletal muscle, BCTA2 activity can be upregulated by exercise, which has been demonstrated by the increase of KIC (2-keto-isocaproate/4-methyl-2-oxopentanoic acid), a subproduct of leucine transamination [2]. Likewise, endurance training has been shown to increase BCAT2 expression in muscle and therefore increases BCAAs oxidation [22]. Physiological regulation of BCTA2 expression seems to be controlled by transcription factors such as PGC-1α factor, which leads to an increase in BCAT expression, likely due to higher mitochondrial activity and via glucocorticoid receptor (GR) [2]. Regarding food intake, it has been shown that BCAAs oxidation is increased after feeding and decreased upon caloric restriction. Interestingly, if fasting is prolonged (starvation), BCAAs oxidation increases again as a feedback mechanism to provide substrates for gluconeogenesis in the liver [26]. In addition, BCAAs oxidation is affected by several factors, e.g., insulin increases BCAAs oxidation [24].

It has been reported that BCKDH is regulated by allosteric inhibition by subproducts (e.g., NADH, α-ketoisocaproate, branched acyl-CoA esters), but it is also regulated by phosphorylation [27]. Regarding BCAAs catabolism in the liver, it is known that BCKDH catabolic enzymes are also positively regulated by transcriptional factors such as PCG-1α and PPAR transcription factor family members in the liver. In contrast, BCKDH activity is decreased by thyroid hormone and during starvation [24]. Furthermore, it is known that BCKAs oxidation might also affect liver metabolism and glucose homeostasis [14]. For instance, BCKAs accumulation caused by decreased BCKA oxidation results in gluconeogenesis inhibition. This interplay between BCAAs and liver glucose metabolism might be explained by the role of the liver as a sensor of BCKAs circulating levels and of BCAA catabolism [13]. Moreover, recent evidence suggests that BCKDH activity can decrease during metabolic alterations (e.g., obesity) leading to a shift in BCKA oxidation from the liver to skeletal muscle and resulting in insulin resistance [28].

These mechanisms might explain in part the evidence of the association between BCAAs and metabolic dysfunction. In fact, increased levels lead to metabolic alterations through mechanisms such as mitochondrial dysfunction and insulin resistance by the activation of the mTOR signaling pathway [29]. Moreover, it has been shown that BCAAs catabolism alterations are associated with the development of CLD [16].

In summary, the catabolism of BCAAs involves intricate processes primarily in the liver, yet significantly influenced by skeletal muscle activity. Regulatory mechanisms, such as exercise, starvation and transcription factors, are fundamental in this process. Dysregulation of BCAAs catabolism may precipitate metabolic dysfunction, underscoring its relevance to CLD.

## 3. Clinical Insights into CLD: Role of BCAAs in Musculoskeletal Health

### 3.1. BCAAs Concentration in Patients with CLD

In advanced stages of cirrhosis, several factors contribute to a poor nutritional state, including low nutrient intake, altered metabolism, impaired absorption, impaired digestion, and rapid satiety. A deficient nutritional status is prevalent among adults with CLD, with reports indicating rates from 5% to 74%, depending on the assessment method used [30]. Among these patients, up to 59% exhibit moderate to severe malnutrition, with alcohol-associated liver disease (ALD) being the most prevalent etiology [31].

As the disease progresses, extended fasting periods and insulin resistance prompt muscle cells to initiate gluconeogenesis. This process utilizes BCAAs as a source for synthesizing glutamine and alanine. Furthermore, skeletal muscles play a role in detoxifying ammonia by transforming ammonia and glutamate into glutamine by glutamine synthetase, a reaction that utilizes BCAAs as the primary nitrogen source. These mechanisms, coupled with malnutrition, collectively contribute to the observed plasmatic depletion of BCAAs in patients with CLD. It has been described (Table 1) that healthy subjects typically exhibit total BCAAs plasma concentrations ranging from 423 μmol/kg to 646 μmol/kg, while patients with cirrhosis of different etiologies (ALD, viral hepatitis, metabolic dysfunction-associated steatotic liver disease (MASLD)) and HCC often demonstrate lower levels, ranging from 278 μmol/kg to 535 μmol/kg across various studies. The studies by Montanari A. et al. [32] and Dam G. et al. [33,34] reported statistically significant differences in Leu and Val concentrations between adults with CLD and healthy individuals. The intracellular levels of BCAAs in muscle and their uptake into muscle are also compromised. Dam G. et al. [33,34] demonstrated a consistent decrease in BCAAs uptake in adults with CLD (67 μmol/min) compared to controls (110 μmol/min). Montanari A. et al. [32] reported a substantial depletion of Val levels in muscle tissue among adults with CLD. Although Val was significantly reduced (222 umol/kg vs. control (368 umol/kg), a comparable declining trend was observed with Leu and Ile levels.

The decline in nutritional status correlates with the progression of liver diseases. Janota B. et al. [40] investigated 118 adults with CLD and demonstrated a strong correlation between the Child-Pugh (CP) score and nutritional status. This deterioration primarily affected muscle mass, falling below standard values in 1.9% (CP score A), 29.4% (CP score B), and 56.2% (CP score C) of cases. Trillos-Almanza. et al. [38] reported a correlation between plasma BCAAs concentrations and the CP score, showing reduced BCAAs levels in more severe cases (CP score B and C) compared to the less severe category (CP score A).

In conclusion, the intricate relationship between CLD, nutritional status, and BCAAs highlights the need for comprehensive nutritional interventions in the management of these patients. Understanding these complex interactions could pave the way for targeted nutritional strategies aimed at improving the wellbeing of individuals with end-stage liver disease, particularly in addressing the challenges associated with malnutrition and BCAAs depletion.

### 3.2. Correlation of Muscle Health and BCAAs in Patients with CLD

The relation between nutritional status, muscle functionality (measured by handgrip strength), and muscle mass (determined by the skeletal muscle index (SMI)) is connected to the development of sarcopenia as demonstrated by Sehgal P. et al. [41]. 

Sarcopenia is currently recognized as muscle failure, defined by a combination of factors such as diminished skeletal muscle quantity, increased fat accumulation within the muscle, reduced muscle strength, compromised physical performance, and alterations in circulating biological markers [42], with low strength as the key characteristic for the condition [43]. In a multicenter study evaluating patients with cirrhosis of diverse etiologies, the prevalence of sarcopenia was 45.4% to 70% [44]. Notably, this prevalence is considerably higher when compared to the 10–16% range observed in the elderly population [45].

Regardless of the nutritional status, patients with CLD experience reduced skeletal muscle strength and mass. Skeletal muscle mass requires the analysis of a group of muscles within a single anatomic area using one or more techniques such as dual-energy X-ray absorptiometry (DXA), bioelectrical impedance analysis (BIA), CT scan or creatinine excretion. Muscle strength evaluation employs dynamometers for isometric strength and measures power and torque for isokinetic strength. Complementary physical performance tests, including gait speed, chair stands, balance tests, the 400 m walk test, and the 6 min walk test, are commonly employed [46]. The association between lower BCAAs levels and diminished muscle strength and functionality has been highlighted by Trillos-Almanza et al. [38]. In a cross-sectional study, 92 CLD patients were submitted to different functional tests that evaluate mobility, agility and muscle strength of different group of muscles. Hand grip strength demonstrated associations with Leu and Ile in males. In more complex tests that evaluate strength, coordination and balance, such as the Timed Up and Go (TUG) and sit-to-stand test, there was an inverse relationship between the test performance and total BCAAs. These findings underscore the association of BCAAs with liver disease severity and impaired muscle function. Xiang Q. et al. [35] corroborated these findings by analyzing hand grip strength in 127 CLD patients with low BCAAs levels, observing a decrease in performance (handgrip strength ≤ 25.47 ± 5.84 kg) and diagnosing sarcopenia in 37 of these patients. These findings underscore the association of BCAAs with liver disease severity and impaired muscle function.

### 3.3. Interplay of BCAAs, mTOR Signaling, Ammonia, and Mitochondrial Dysfunction in the Skeletal Muscle during CLD

The mechanisms underlying muscle mass loss and functionality have been extensively studied. Skeletal muscle mass equilibrium depends on factors such as protein intake, synthesis, and degradation. Many factors contribute to low nutrient intake, including dietary restrictions, altered satiety (linked to low ghrelin and high leptin plasma levels [47], substance abuse (opioids, alcohol), and HE. Regarding protein degradation, this one is impaired in chronic liver disease since endoplasmic stress activates the ubiquitin-proteasome system (UPS), which cause protein misfold and autophagy, a cellular process crucial for the degradation and recycling of cellular components. On the other hand, among the key signaling pathways associated with nutritional and metabolic status is the mTOR pathway. BCAAs play a crucial role in protein synthesis and mTOR1 signaling.

mTOR signaling is crucial due to its role in regulating various metabolic processes, including ureagenesis. Impaired mTOR signaling disrupts ureagenesis, leading to increased ammonia levels, particularly notable in adults with cirrhosis and sarcopenia where venous ammonia levels are elevated [48]. Elevated ammonia levels can prompt increased ammonia uptake in muscles, resulting in the overactivation of the TCA cycle. This overactivation, in turn, reduces mitochondrial ATP synthesis, ultimately triggering a senescence-associated secretory phenotype in skeletal muscle. This phenomenon is mediated by sirtuin-mediated deacetylation [49,50]. Furthermore, it contributes to heightened oxidative stress within the muscles, as suggested by Davuluri, G. et al. [51]. Hence, maintaining functional mTOR signaling helps to regulate these processes and BCAAs may play a role in mitigating the adverse effects associated with increased ammonia levels and oxidative stress in skeletal muscles.

Skeletal muscle from patients with low BCAAs levels and cirrhosis reveals reduced the activation of downstream targets of mTOR1 (p70S6K, S6, 4EBP1) but elevated levels of the intracellular amino acid sensor General Control of Nutrition Derepressed 2 (GCN2) [37]. This suggests activation of autophagic pathways, as GCN2 is known to elevate autophagic activity in response to short-term essential amino acid deprivation, particularly leucine, in an mTOR1 dependent manner [52].

Additionally, ammonia levels among patients with CLD range from 57.7 ± 30.1 µg/dL [36] to 125 ± 50 µg/dL [32], while healthy adults maintain concentrations between 19–94 µg/dL, varying between genders [53]. This high ammonia concentration correlates with what was found by Dam G. et al. [33], who demonstrated lower BCAAs uptake in leg muscles in adults with CLD (healthy: 196 ± 67 µmol/L vs. cirrhosis: −84.7 ± 110 µmol/L), with those of ALD etiology exhibiting the most pronounced effects [34]. This diminished uptake adversely affects muscle ammonia detoxification.

Ammonia serves as a stimulant for proteolysis in adults with CLD by initiating NF-κB-mediated transcription of myostatin [54]. Elevated myostatin levels, triggered by ammonia, further escalate proteolysis via Akt inhibition and autophagy activation [55]. Conversely, BCAAs are known to inhibit proteolysis [56,57], and one of the mechanisms involved is ubiquitin-proteasome signaling involving TRIM63 (MURF1) and FBXO32 (Atrogin-1) [58]. In a study by Tsien C. et al. [37] involving muscle samples from ALD cirrhotic patients (n = 6), increased myostatin protein were observed, accompanied by BCAAs depletion (Table 1).

Finally, high ammonia increases the TCA cycle causing mitochondrial dysfunction in muscle cells. In this matter, Doi J. et al. [39] studied patients (n = 8) with cirrhosis of viral etiology and a observe low (0.68–2.31) Fischer´s ratio (BCAAs/AAAs) (normal ≥ 3) together with a significantly change in the intracellular pH (from 7.35 to 0.49 ± 0.16 compared to 0.20 ± 0.18 in healthy individuals) and the creatine phosphate index (0.55 ± 0.12 compared to 0.35 ± 0.19 in the healthy volunteers) in skeletal muscle samples. Both of these changes indicate an overwork in energy production and metabolism, suggesting an indirect link between low BCAAs and altered mitochondrial function, which could explain the impaired protein synthesis and muscle functionality.

**Figure 2:**
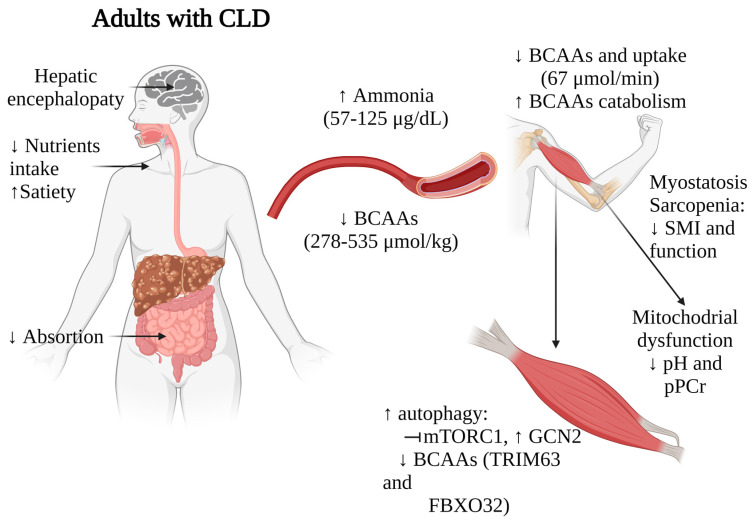
Impact of BCAAs alterations on muscle functionality in adults with CLD. Arrows indicate increase (↑) or decrease (↓). CLD, chronic liver disease; BCAAs, branched-chain amino acids. Figure created with Biorender.com (accessed on 13 January 2024).

Taken together, these findings suggest that BCAAs depletion, along with alterations in ammonia metabolism, myostatin activation, and disrupted mTOR signaling, significantly impact muscle mass, functionality, and overall nutritional status in patients with liver diseases (Figure 2). These multifaceted relationships underscore the complex interplay between liver function, muscle metabolism, and nutritional status in these individuals. While further research and validation studies are necessary to establish the value of BCAAs levels as prognostic markers, the strong correlations observed in multiple studies indeed suggest their potential utility in staging nutritional status and identifying sarcopenia in patients with CLD.

## 4. Interventions with BCAAs Supplementation in Patients with CLD: Implications for the Skeletal Muscle

Oral BCAAs supplementation with BCAAs-enriched formulas is often recommended by international guidelines in the management of cirrhosis-related complications [59,60], mainly HE and impaired nutritional status. Some studies on BCAAs supplementation have demonstrated improvements in clinical indicators such as the CP score, MELD score, lower incidence of HCC and better survival rates [61,62,63,64]. Substantial evidence suggests that BCAAs supplementation not only influences cirrhosis-related parameters but also has positive effects on skeletal muscle function parameters, making it a valuable intervention in the management of complications of CLD (Table 2). In this section we will discuss key findings from studies investigating the impact of BCAAs supplementation on muscle health in patients with CLD.

### 4.1. Effects of BCAAs on Skeletal Muscle Mass and Skeletal Muscle Strength

Studies on the effects of BCAAs supplementation on skeletal muscle parameters in patients with CLD reveal promising outcomes. Ruiz-Margáin A. et al. [65] conducted a randomized controlled trial (RCT) to study the effect of BCAAs supplementation (110 g/day) on nutritional status. The study reported increased skeletal muscle mass, as indicated by mid-arm muscle circumference (MAMC) (from 28.7 ± 5.3 cm to 30.5 ± 4.6 cm, *p* = 0.000), and a decrease in the triceps skinfold (TSF) in the BCAAs group (from 21.1 ± 12.2 mm to 19.6 ± 7.5 mm, *p* = 0.000). This is in line with the findings reported by Singh Tejavath A. et al. [66] in an RCT, on BCAAs supplementation (7.2 g/dose). In this study, significant improvements were reported in skeletal muscle mass, determined by total abdominal muscle area (TAMA), TSF, and MAMC (*p* = 0.001, *p* = 0.039, and *p* = 0.03, respectively) and in muscle function, evaluated with the improvement of the hand-grip strength score before and after the intervention (from 23.79 ± 5.28 kg to 25.94 ± 5.14 kg, *p* = 0.000), as well as the gait speed score before treatment (0.83 ± 0.07 ms^−1^) and after 6 months of treatment (1.12 ± 0.04 ms^−1^). These studies suggest that BCAAs play a crucial role in preserving skeletal muscle health in CLD patients. Les I. et al. [67] conducted a RCT involving adults with CLD and a history of HE, in which oral supplementation with BCAAs (30 g twice a day) was compared to maltodextrin. The 14-month intervention revealed increased MAMC in the BCAAs group. However, there was no significant difference in the risk of remaining free of HE among the groups (*p* = 0.274).

**Table 2:** Muscle-related parameters in patients with CLD receiving BCAAs supplementation.

Reference	Type of Study	Intervention	Treatment Composition	Inclusion Criteria	Sample Size	Treatment Duration	Findings
[35]	Open-label prospective study.	Oral supplementation with BCAAs (4.5 g/day) as a late evening snack, compared to a combination of BCAAs supplementation with unsupervised walking exercise, or unsupervised walking exercise alone.	BCAAs 4.5 g, containing: Leu 3 g Ile 0.75 g Val 0.75 g	CLD with CP score A or B	127 patients: BCAAs group, n = 42 Walking exercise, n = 43 Walking exercise plus BCAAs, n = 42	3 months.	-Walking exercise plus BCAAs significantly increased grip strength and SMI, reducing sarcopenia prevalence. -Albumin significantly increased in all groups.
[68]	RCT.	Oral supplementation with BCAAs (10.85 g/day) against regular diet.	BCAAs 5.425 g, containing: Leu 2.03 g Ile 1.76 g Val 1.635 g	CLD in frail patients with CP score A or B	54 patients (27 in the BCAAs group).	4 months.	-Increased SMI in the intervention group. -Improvement in all 4 domains of the physical component score of the SF-36 questionnaire in the BCAAs group. -Improvement in the LFI, BMI and serum albumin compared to controls.
[69]	RCT.	Oral supplementation with BCAAs (12 g/day) against placebo, in adition to a home-based exercise program (30 min/day), dietary counselling and standard medical therapy.		CLD with CP score A or B and sarcopenia	60 patients (30 in the BCAAs group).	6 months.	No changes in muscle mass with the addition of BCAAs to exercise, dietary counselling and standard medical therapy.
[66]	RCT.	Oral supplementation with BCAAs (7.2 g/dose) against lactoalbumin (6.3 g/dose).	BCAAs 8.1 g, containing: Leu 1.2 g Ile 0.6 g Val 0.6 g	CLD, with portal hypertension and sarcopenia	138 patients (69 in the BCAAs group)	6 months.	-Improvement of the hand grip strength. -Improvement of the gait speed score from before treatment with BCAAs to after treatment. -Increased in the TAMA, TSF, and MAMC. -Lower progression of HE.
[70]	RCT.	Oral supplementation with BCAAs (5.24 g/day) and physical activity against nutritional and physical activity intervention.	Each 100-g sachet contained: Leu 2.61 g Ile 1.01 g Val 1.62 g	CLD and sarcopenia	32 patients (15 in the BCAAs group)	3 months.	-Improvement in muscle mass and serum albumin levels compared to controls. -Improvement in the LFI in the global cohort.
[36]	Cohort study.	Oral supplementation with BCAAs (4 g/dose) three times daily.	BCAAs 4 g, containing: Leu 1.9 g Ile 0.9 g Val 1.14 g	CLD with serum albumin levels of ≤3.5 g/dL	21 patients.	12 months.	-Decreased IMAC in patients with ameliorated hypoalbuminemia associated with BCAAs supplementation. -Higher liver-related event-free survival rates in patients with decreased IMAC.
[65]	RCT.	Oral supplementation with BCAAs (110 g/day) against high-fiber and high-protein diet.	BCAAs 110 g, containing: Leu 3.38 g Ile 2.75 g Val 2.5 g	CLD and CP score A or B	72 patients (73 in the BCAAs group)	6 months.	-Increased muscle mass and decreased fat mass compared to control. -No differences in ammonia and glucose levels. -No significant changes in the Psychometric HE Score or the Critical Flicker–Frequency score results.
[37]	RCT.	Single oral BCAAs mixture (15 g) enriched with Leu, and a primed constant (0.05 µmol.kg^−1^.min^−1^) infusion of L-[ring-2H5]-phenylalanine.	BCAAs 15 g, containing: Leu 7.5 g Ile 3.75 Val 3.75 g	CLD due to ALD, with CP score A, and alcohol abstinence for at least 6 months	6 patients. 8 controls.	7 h.	-BCAAs/Leu acutely reversed impaired mTOR1 signaling and increased autophagy in skeletal muscle of cirrhotic ALD patients. -Plasma BCAAs increased similarly in cirrhotic patients and controls after oral intake. -Muscle synthesis rate was slightly higher in controls. -BCAAs/Leu supplementation reduced elevated protein breakdown in cirrhosis patients.
[67]	RCT.	Oral supplementation with BCAAs (30 g) or maltodextrin twice a day.	BCAAs 30 g, containing: Leu 13.5 g Ile 9 g Val 7.5 g	CLD and a previous episode of HE	116 patients (58 in the BCAAs group).	14 months.	-Increased MAMC in the BCAAs group. -No difference in the risk of remaining free of HE among groups.

Others studied the effect of BCAAs on serum albumin levels as a marker of liver disease severity. Kitajima Y. et al. [36] investigated the impact of BCAAs supplementation (4 g/dose, three times daily) over a 12 month period in patients with hypoalbuminemia. The cohort study revealed a decrease in intramuscular adipose tissue content (IMAC), particularly in patients with improved hypoalbuminemia. Furthermore, skeletal muscle mass was maintained in patients with improved hypoalbuminemia (12.4 ± 3.0 to 12.5 ± 3.2 cm^2^/m^2^, *p* = 0.4236) in comparison to patients that remained hypoalbuminemic (12.3 ± 2.3 to 11.5 ± 2.4 cm^2^/m^2^, *p* = 0.001), emphasizing the potential of BCAAs to prevent the progression of sarcopenia associated with liver cirrhosis. The observed beneficial effects of BCAAs supplementation on serum albumin levels suggests a multifaceted role of BCAAs in preserving both skeletal muscle and hepatic function.

### 4.2. Role of BCAAs in Frailty and Nutritional Status

Siramolpiwat S. et al. [68] conducted a 4-month RCT involving frail CLD patients with CP scores A or B that compared oral supplementation with BCAAs (10.85 g/day) with a regular diet. The primary outcome, frailty reversion, was significantly higher in the BCAAs group at week 16 (Liver Frailty Index (LFI) −0.36 ± 0.3 vs. −0.15 ± 0.28, *p* = 0.01), with notable improvements in SMI, the physical component score of the SF-36 questionnaire, BMI, and serum albumin level. A closer examination of the results revealed a trend towards higher frailty reversion as early as week 8, reinforcing the notion that BCAAs play an important role in mitigating frailty in this patient population. The improvement observed in frailty with BCAAs supplementation aligns with the findings from the controlled trial of Hernández-Conde M. et al. [70] in adults with CLD and sarcopenia. Over a 3-month period, this study demonstrated significant improvements in skeletal muscle mass and serum albumin levels in the BCAAs group compared to controls. The LFI showed a noteworthy reduction in both groups (LFI for the global cohort 4.2 vs. 3.9; *p* < 0.001), emphasizing the potential benefits of an adequate nutritional intervention on frailty amelioration.

### 4.3. Role of BCAAs on Skeletal Muscle Protein Metabolism

The RCT by Tsien C. et al. [37], using a single oral dose of a BCAAs mixture enriched with Leu (BCAAs/Leu), sheds light on the molecular perturbations responsible for sarcopenia in adults with CLD due to ALD (n = 6): BCAAs/Leu supplementation reversed the impairment of mTOR1 signaling and increased autophagy in the skeletal muscles of those patients. This finding is particularly significant, as Leu was shown to directly activate mTOR1, leading to enhanced protein synthesis while simultaneously mitigating protein breakdown.

Additionally, the reduction in autophagy markers (lipidation of LC3, ATG 5,7 expression, P62 degradation and Beclin1 overexpression) following BCAAs/Leu administration demonstrates that this supplementation modulates skeletal muscle protein breakdown in adults with CLD and that autophagy, rather than the ubiquitin-proteasome system, is primarily responsible for skeletal muscle proteolysis in cirrhosis. The suppression of autophagy by BCAAs/Leu highlights a potential avenue for therapeutic intervention to mitigate skeletal muscle protein breakdown in adults with cirrhosis secondary to ALD.

### 4.4. Combining BCAAs with Exercise

Aerobic exercise increases the muscle protein synthesis, mitochondrial biogenesis and dynamics, contributes to the restoration of mitochondrial metabolism and reduces the expression of genes involved in catabolism (FOXO3a, MuRF-1, myostatin) [71,72]. In patients with CLD, exercise is considered an important strategy for ameliorating sarcopenia as it prevents skeletal muscle breakdown and maintains physical function [73]. This is achieved through its role in upregulating insulin-like growth factor 1, which could result in the downregulation of myostatin [73]. In relation to the BCAAs supplementation during physical activity, the study by Xiang Q. et al. [35] underscores the importance of the timing and intensity of exercise interventions. They found that post-exercise, BCAAs increases skeletal muscle mass (*p* = 0.012) and grip strength (*p* = 0.036), and decreases the prevalence of sarcopenia (*p* = 0.015), when compared to the exercise-only group. Similarly, Hernández-Conde M. et al. [70] explored BCAAs supplementation (5.24 g/day) in combination with physical activity in cirrhotic patients with sarcopenia. The 12-week intervention resulted in increased skeletal muscle mass in the BCAAs group (83.3 vs. 46.7%; *p* = 0.056) and improved LFI score. The study highlights the potential of BCAAs to enhance nutritional and physical activity interventions, leading to a positive impact on skeletal muscle mass and serum albumin levels.

However, contrasting results were obtained in a controlled trial with oral BCAAs supplementation (12 g/day) in combination with a home-based exercise program, dietary counseling, and standard medical therapy [69]. In this study, spanning six months and including patients with CLD and sarcopenia, no significant improvement in skeletal muscle parameters (SMI 0.84, 95% CI −2.9, +1.2, *p* = 0.420) or quality of life was observed. Moreover, no changes in serum myostatin and plasma ammonia levels were found in the BCAAs group, suggesting that targeting a single pathway with BCAAs may not be sufficient in this population. The absence of changes in these markers suggests that BCAAs alone may not effectively modulate the underlying molecular pathways contributing to muscle wasting in CLD patients. Furthermore, the authors propose the existence of a ‘ceiling effect’ in the uptake of essential amino acids for skeletal muscle formation. This effect implies that the benefits of dietary BCAAs might be limited, especially in patients with cirrhosis maintaining adequate dietary intake. The study raises the intriguing possibility that these patients may have reached the limit of amino acid uptake required for skeletal muscle formation, rendering additional BCAAs intake ineffective in fostering increased skeletal muscle mass.

Although the initial studies were very promising, follow-up studies demonstrating contrasting results warrant a critical examination of exercise interventions, e.g., duration and intensity as well as the patient population that will benefit from these interventions. 

## 5. Clinical Perspectives

The positive effects of BCAAs supplementation on skeletal muscle mass, strength, and overall nutritional status in cirrhosis are noteworthy. These effects include improvements in muscle protein synthesis, preservation of lean body mass, enhanced exercise performance, and amelioration of muscle-related complications such as sarcopenia. These effects are particularly crucial in cirrhosis, where muscle wasting and malnutrition often occur. By enhancing these physiological parameters, BCAAs supplementation presents a promising avenue for intervention, potentially ameliorating the deleterious consequences of cirrhosis-related complications.

The clinical implications of integrating BCAAs with physical exercise into a precision-oriented approach for cirrhosis management strategies are substantial. By offering personalized interventions, clinicians may effectively mitigate the progression of liver disease and enhance patient outcomes. This approach not only targets the symptoms but also addresses underlying metabolic imbalances, potentially leading to improved quality of life for individuals with CLD. Nevertheless, further research is needed to establish exercise regimes and optimal BCAAs supplementation, including dosage optimization, timing considerations, long-term effects, population specificity, potential synergies with other supplements, mechanisms of action, safety assessments, comparative studies, and real-world applications, as well as to delineate the characteristics of the patient population that will benefit from these interventions. Investigating these factors will refine interventions and improve care for (selected) patients with CLD.

## 6. Conclusions

The beneficial effects of BCAAs on skeletal muscle mass improvement can be attributed to their role as a precursor for protein synthesis and as a preferential energy source in skeletal muscle. Chronic inflammation, impaired nutrient intake, malabsorption, and hormonal changes associated with CLD contribute to sarcopenia. Furthermore, cirrhosis induces a hypermetabolic state, leading to increased fatty acid oxidation and gluconeogenesis, resulting in low plasma BCAAs levels. BCAAs supplementation addresses this metabolic imbalance, supporting muscle strength and mitigating frailty. Additionally, the concept of skeletal muscle–liver crosstalk underscores the intricate relationship between skeletal muscle and liver in BCAAs metabolism, highlighting the potential of targeted interventions to improve both hepatic and muscular outcomes in CLD.

## Figures and Tables

**Table 1:** BCAAs and muscle health during CLD.

Reference	Type of Study	Population	Etiology	BCAAs Analysis	Liver Function	Skeletal Muscle Function
[32]	Comparative study	14 adults with CLD (12 males, 2 females) and 9 healthy controls.	11-ALD 3-cryptogenetic	Spectrophotometry ↑ BCAAs (μM/kg) controls: Leu 132, Val 215, T. BCAAs 423. ↓ BCAAs (μM/kg) patients: Leu 84, Val 54, T. BCAAs 278.	Patients profile: -Albumin (gm/L): 28.6 ± 5.1 -PT (%): 67 ± 14 -Ammonia (µg/dL): 125 ± 50 -Ascites: 7 patients -Malnutrition: 7 patients	Intracellular muscle Val: Patients: 222 μM/kg (↓) Control: 368 μM/kg.
[33]	Cohort design	14 patients with CLD (13 males, 1 female) and 7 healthy subjects (5 males, 2 females).	Non specify	HPLC with fluorescence detection. ↑ BCAAs (μM/kg) controls: Leu 132, Val 215, T. BCAAs 423. ↓ BCAAs (μM/kg) patients: Leu 84, Val 54, T. BCAAs 278.	Patients profile: -Albumin (μmol/L): 520 ± 21 -Total bilirubin (μmol/L): 21.8 ± 3.2-PT index: 0.58 ± 0.03 -CP score: A: 6; B: 8; C: 0 patients. Healthy profile: -Albumin (μmol/L): 639 ± 27 -Total bilirubin (μmol/L): 7.14 ± 1.1 -PT index: 0.94 ± 0.05	Uptake of BCAAs across muscle leg (femoral artery): Control: 196 ± 67 μmol/min Patients: −84.7 ± 110 μmol/min).
[34]	Cohort design, retrospective	13 patients with CLD and 6 patients with an episode of alcoholic hepatitis (16 males, 4 females) and 6 healthy controls (2 females, 5 males)	6-ALD 13-Other	↑ BCAAs (μmol/L) controls: Leu 244 ± 19, Val 256 ± 19, T. BCAAs 646 ± 44. ↓ BCAAs (μmol/L) patients: Leu 185 ± 18, Val 201 ± 15, T. BCAAs 535 ± 42.	ALD patients profile: -Albumin (μmol/L): 469 ± 42 -Total bilirubin (μmol/L): 380 ± 42 -PT index: 0.24 ± 0.04 -CP score: A: 0; B: 2; C: 4 patients. CLD patients profile: -Albumin (μmol/L): 520 ± 21 -Total bilirubin (μmol/L): 21.8 ± 3 -PT index: 0.58 ± 0.03 -CP score: A: 6; B: 7; C: 0 patients	Leg muscle BCAAs uptake: Patients with alcoholic hepatitis: Total BCAAs 0.48 ± 23 μmol/L Stable cirrhosis: Total BCAAs 32 ± 22 μmol/L Controls: Total BCAAs −11.9 ± 22 μmol/L
[35]	Prospective control trial	127 patients with CLD (81 males, 43 females)	68-HBV 23-ALD 26-MASLD 7-Other	Colorimetric method Average of BCAAs 453.73 +/− 40.17 (μmol/L)	Patients profile: -Albumin (g/dL): 32.48 ± 4.53 -Total bilirubin (μmol/L): 36.65 ± 13.16 (μmol/L) -PT (%) 62.48 ± 10.45 -CP score: A: 71; B: 53 patients.	Patients had decreased handgrip strength (kg): 25.47 ± 5.84 and SMI (cm^2^/m^2^): 49.61 ± 8.84. 37 patients with sarcopenia.
[36]	Case control	21 patients with CLD (9 males, 12 females)	18-HCV 2-HBV 1-ALD	Patients with Albumin improvement (11): ↓ Total BCAAs 377.4 ± 102.2 μmol/L Patients with no Albumin improvement (10): ↑ Total BCAAs 413.4 ± 89.2 μmol/L.	Patients profile: -Albumin (g/dL): 3.2 ± 0.4 -Total bilirubin (mg/dL): 1.2 ± 0.5 -Ammonia (μg/dL) 57.7 ± 30.1 -CP score: A: 10; B: 11; C: 12 patients.	SMI area: 12.4 ± 2.7 cm^2^/m^2^. IMAC: −0.11 ± 0.16.
[37]	Control trial	8 healthy controls (4 males, 4 females). 6 patients with CLD (5 males, 1 female).	ALD cirrhosis	Ion exchange chromatography ↑ BCAAs (μmol/L) controls: Leu 119.13 ± 9.35, Ile 77.02 ± 5.78, Val 245.70 ± 18.69. ↓ BCAAs (μmol/L) patients: Leu 81.33 ± 7.75, Ile 55.15 ± 4.12, Val 166.99 ± 12.44.	Patients profile: -Albumin (μmol/L): 4.20 ± 0.12 -CP score: 5.17 ± 0.15 -MELD score: 6.21 ± 0.23 -Ammonia (μg/dL) 98.24 ± 6.75	Patients muscle tissue: -Lower activation of mTOR-related proteins (p70S6K, S6, 4EBP1) -↑ Intracellular amino acid sensor (GCN2) and myostatin protein.
[38]	Cross-sectional, single-center analysis.	92 patients with CLD (60 males, 32 females).	7-Viral hepatitis 24-Autoimmune 28-MASLD 20-ALD 2-Storage disorder 3-Malignancy 8-Other	NMR spectroscopy platform Control group: ↑ T. BCAAs (μmol/L): -Men: 412.7 -Women: 339.7 Patients group: ↓ T. BCAAs (μmol/L): -Men: 307.0 -Women: 213.5	Patients profile: -Albumin (g/L): 32.7 ± 6.5 -Total bilirubin (µmol/L): 41 -Ammonia (µmol/L) 68.8 ± 33.4 -MELD score 15 ± 6.	-Lowest BCAAs tertile had higher CP scores (7 ± 2) -The 4 m walking test, standing balance test and CFS were not significantly different between BCAAs tertiles -Inverse correlations of the Timed up and go performance test with total BCAAs, Val and Ile. -Total BCAAs, Val and Leu were inversely related in the sit-to-stand test
[39]	Comparative study	8 healthy subjects (6 males, 2 females) and 8 patients with CLD (4 males, 4 females)	6-HCV 1-HBV 1-Other	Patients group: Fischer’s ratio 0.68–2.31	Patients profile: -Albumin (g/dL): 2.8–3.4 -Total bilirubin (mg/dL): 0.6–2.1 -Ammonia (µg/dL): 46–122 -PT (%): 58–77	After exercise loading intramuscular ⊿pH: -Controls 0.20 ± 0.18 -Patients 0.49 ± 0.16 Creatine phosphate: -Controls 0.35 ± 0.19 -Patients 0.55 ± 0.12

Arrows indicate increase (↑) or decrease (↓) in concentrations.

## Data Availability

The data presented in this study are available upon request from the corresponding author.

## Abbreviations

ALD	alcohol-associated liver disease.
BCAAs	Branched-chain Amino Acids.
BCAT	branched-chain aminotransferase.
BCKAs	branched-chain α-ketoacids.
BCKDH	branched-chain α-keto acid dehydrogenase.
CFS	Clinical Frailty Scale.
CLD	Chronic Liver Disease.
HBV	hepatitis B virus.
HBV	hepatitis C virus.
HCC	hepatocellular carcinoma.
HPLC	high-performance liquid chromatography.
Ile	isoleucine.
IMAC	intramuscular adipose tissue content.
Leu	leucine.
LFI	liver frailty index.
MAMC	mid-arm muscle circumference.
MASLD	Metabolic Dysfunction-Associated Steatotic Liver Disease.
MELD	Model for End-Stage Liver Disease.
mTOR	mammalian target of rapamycin.
NMR	Proton (1H) nuclear magnetic resonance.
PT	prothrombin time.
RCT	randomized controlled trial.
SMI	skeletal muscle mass index.
TAMA	total abdominal muscle area.
TCA	citric acid cycle.
TSF	triceps skinfold.
Val	valine.
